# New Data and New Features of the FunRiceGenes (Functionally Characterized Rice Genes) Database: 2021 Update

**DOI:** 10.1186/s12284-022-00569-1

**Published:** 2022-04-19

**Authors:** Fangfang Huang, Yingru Jiang, Tiantian Chen, Haoran Li, Mengjia Fu, Yazhou Wang, Yufang Xu, Yang Li, Zhengfu Zhou, Lihua Jia, Yidan Ouyang, Wen Yao

**Affiliations:** 1 National Key Laboratory of Wheat and Maize Crop Science, College of Life Sciences, Henan Agricultural University, Zhengzhou, 450002 China; 2 Henan Academy of Crop Molecular Breeding, Henan Academy of Agricultural Sciences, Zhengzhou, 450002 China; 3 National Key Laboratory of Wheat and Maize Crop Science, College of Agronomy, Henan Agricultural University, Zhengzhou, 450002 China; 4 National Key Laboratory of Crop Genetic Improvement, National Center of Plant Gene Research, Huazhong Agricultural University, Wuhan, 430070 China

**Keywords:** Rice, *Oryza sativa*, Functionally characterized genes, Functional genomics, Database

## Abstract

As a major food crop and model organism, rice has been mostly studied with the largest number of functionally characterized genes among all crops. We previously built the funRiceGenes database including ~ 2800 functionally characterized rice genes and ~ 5000 members of different gene families. Since being published, the funRiceGenes database has been accessed by more than 54,400 users with over 540,000 pageviews. The funRiceGenes database has been continuously updated with newly cloned rice genes and newly published literature, based on the progress of rice functional genomics studies. Up to Nov 2021, ~ 4100 functionally characterized rice genes and ~ 6000 members of different gene families were collected in funRiceGenes, accounting for 22.3% of the 39,045 annotated protein-coding genes in the rice genome. Here, we summarized the update of the funRiceGenes database with new data and new features in the last 5 years.

## Background

Rice (*Oryza sativa*) is a major food crop for almost half of the world population. Identification of functional genes governing the phenotypes of complex agronomic traits in rice is crucial to safeguard the world’s food security. Rice is also a model organism for plant genomics researches, benefiting from its small genome size with accurate genome sequence and a great number of molecular markers, as well as the availability of high-efficiency transgenic systems. Most studies on the functions of rice genes can be directly applied to homologous genes in other cereals and crops. Accordingly, it is important for functional studies and genetic improvement of rice and other crops to collect and build a detailed archive of all functionally characterized rice genes. We previously constructed funRiceGenes, a database that provides the most detailed information of ~ 2800 functionally characterized rice genes (Yao et al. 2017). The data of funRiceGenes database was collected by integrating data extracted from published literature on rice functional genomics deposited in PubMed, the Rice Genome Annotation Project database (Kawahara et al. 2013), the China Rice Data Center (https://www.ricedata.cn/) and the Oryzabase database (Yamazaki et al. 2010). Since being released, this database has been continuously updated based on the latest research progress in rice. Up to November 2021, over 4100 functionally characterized rice genes and more than 6000 gene family members were collected in the funRiceGenes database, providing an important resource for functional genomics research and molecular marker-assisted breeding in rice. In addition, new features were developed for better user experiences and deeper utilization of the funRiceGenes database.

## Results

### Application of the FunRiceGenes Database in Rice and Other Crops

**Fig. 1:**
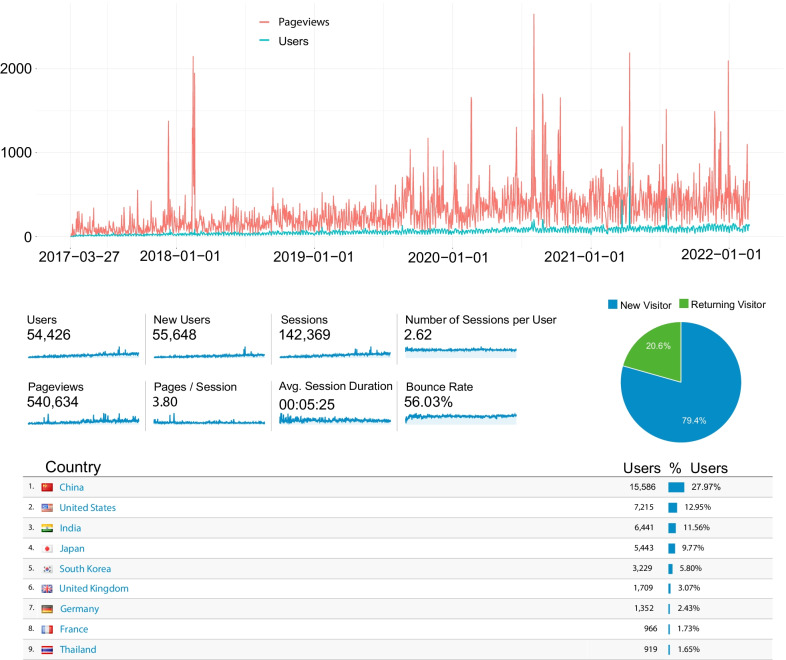
Statistics of the funRiceGenes database visits reported by Google Analytics. The top panel displays the daily user visits and pageviews of the funRiceGenes database from Mar 27 2017 to Feb 24 2022. The middle panel lists the total user visits, pageviews, and other statistics in the last 5 years. The bottom panel shows the proportion of user visits from different countries in the last 5 years

Since being published in December 2017, the funRiceGenes database has been visited by more than 54,400 users, with more than 142,000 sessions and over 540,000 pageviews (Fig. 1) (Yao et al. 2017). The daily number of users and pageviews of the funRiceGenes database were steadily rising in the last 5 years. An average of 3–4 pages was accessed per session. In 2021, the funRiceGenes database was visited by an average of 1917 users per month, with a maximum of 2519 user visits in July. We observed two significant peaks in the daily pageviews of the funRiceGenes database on December 11, 2017 and February 14, 2018, corresponding to the advance online date and formal publishing date of our previous publication in GigaScience (Fig. 1) (Yao et al. 2017). We also observed other significant peaks, which were probably caused by high-impact publications citing the funRiceGenes database or advertisement of funRiceGenes in plenary talks of prominent experts in the rice community (Wing et al. 2018; Li et al. 2021). The top three most visited pages were the GENE, FAMS and DOCS pages of https://funricegenes.github.io/, which listed all the genes, gene families, published papers deposited in the funRiceGenes database, respectively. Apart from the three pages, the top 10 most visited pages were OsPht1, OsHKT1, IPA1, ZYGO1, Gn1a, OsWRKY45, ERF, WRKY, OsTIR1, SD1. The pages of ERF and WRKY itemize all the members of the ERF and WRKY gene families, while the other eight pages record the detailed information of eight notable genes in rice (Rice Wrky Working Group 2012; Nakano et al. 2006). *Ideal Plant Architecture 1* (*IPA1*) is the first gene identified in rice that can promote yield and disease resistance at the same time by maintaining the balance between growth and disease resistance (Wang et al. 2018). Most of the sessions were from countries including China, the United States, India, Japan, and South Korea. To be noted, users from China, India, Japan, and South Korea accounted for more than 50% of all accessions, consistent with the reality that rice is the main food in Asia. Nevertheless, visits from the United States accounted for 13.3% of all accessions, ranking second among all countries. An in-depth investigation revealed that the funRiceGenes database was frequently visited by several cities including Beijing, New Delhi, Hangzhou, Wuhan, Seoul, and Shanghai, which was probably attributed to well-known rice research institutions located in these cities, including the Institute of Genetics and Developmental Biology in Chinese Academy of Sciences in Beijing; the China National Rice Research Institute in Hangzhou; the National Key Laboratory of Crop Genetic Improvement in Huazhong Agricultural University in Wuhan; the Institute of Plant Physiology and Ecology in Chinese Academy of Sciences in Shanghai, etc.

As of February 2022, the funRiceGenes database and the corresponding publication in *GigaScience* have been cited by 102 times (Yao et al. 2017). funRiceGenes provides the most accurate number of functionally characterized rice genes, which was referenced by many studies (Wing et al. 2018; Yang et al. 2020). The datasets in funRiceGenes were utilized to train models in bioinformatics studies (Gupta et al. 2021). The information of more than 4100 genes collected in funRiceGenes was frequently utilized to annotate the functions of gene sets obtained in many studies in rice (Kim et al. 2020; Qin et al. 2021; Zhang et al. 2021). Furthermore, the funRiceGenes database was also used to disclose the potential functions of homologous genes in non-model plants including wheat, rye, sorghum, barley and pacaya palm (Hosni et al. 2021; Pang et al. 2020; Li et al. 2021; Wittern et al. 2021; Dhaka et al. 2020; Wang et al. 2021).

### Features Update of the FunRiceGenes Database

A static website (https://funricegenes.github.io/) was developed for browsing and searching functional rice genes deposited in funRiceGenes. Eight menus were deployed in the static website, including HOME, GENE, FAMS, KEYS, NEWS, DOCS, CITE, and LINK. The symbols of more than 4100 functionally characterized rice genes are listed in the GENE menu. Detailed information of a gene can be viewed by clicking on the corresponding gene symbol, including published articles related to the gene, MSU and RAPdb genomic locus of the gene (Kawahara et al. 2013; Sakai et al. 2013), GenBank accession number, key information on the function of the gene, and information of related genes. The information of more than 6000 gene family members was recorded in the FAMS menu. Information of all members and related publications can be viewed by clicking the name of a specific gene family. Based on the published literature, we extracted more than 400 commonly used keywords concerning various functions of rice genes. All the keywords and the genes related to each keyword can be viewed in the KEYS menu, allowing rapid identification of all the functionally characterized genes related to a specific agronomic trait or keyword. The detailed updating records of the funRiceGenes database since being released in 2014 can be found in the NEWS menu. More than 7000 literatures on rice functional genomics studies are listed in the DOCS menu. To promote the usage of funRiceGenes, publications citing the funRiceGenes database are listed in the CITE menu. The LINK menu provides links to some useful plant databases and bioinformatics applications. Finally, key information including gene list, gene family list, keywords list, and other datasets collected in funRiceGenes can be downloaded from the HOME page of the database. To further facilitate the convenient usage of the static website, in-site searching was enabled with the help of Google and Bing, which were placed on the HOME page of the static website. Moreover, we utilized Bioschemas to install 590 keywords related to rice functional genomics in the metadata of the static website, aiming to facilitate the findability of the website by search engines (Michel 2018). Real-time visitor statistics of the static website since Oct 29 2021 recorded by RevolverMaps (https://www.revolvermaps.com/) is also displayed on the HOME page.

We further developed an R/Shiny web application for interactive queries of the funRiceGenes database in the previous study (Jia et al. 2021; Yao et al. 2017). The URL of the interactive web application was moved from http://funricegenes.ncpgr.cn/ to https://venyao.xyz/funRiceGenes/. In addition to query by gene symbols or keywords, the web application can be searched by the MSU or RAPdb genomic locus. In this study, we developed a new functionality under the ‘Download’ menu of the web application, which can be utilized to batch retrieve data from the funRiceGenes database by multiple user-input MSU or RAPdb genomic loci, or a user-input genomic region. The IDConversion functionality of the web application can be used to perform the conversion between MSU and RAPdb genomic locus, as well as the identification of orthologous genes between *indica* and *japonica* rice. To enable searching of the genic sequences, CDS, or protein sequences of all collected functional genes by sequence similarity, a BLAST interface was deployed in the web application, in this study. To further expand the application of the datasets collected in funRiceGenes, we developed a new feature under the ‘Annotation’ menu of the web application for functional annotation of gene sets obtained in experimental or high-throughput studies in rice. For an input gene set, a word cloud would be created utilizing key messages on the functions of input genes deposited in funRiceGenes.

### Data Update of the FunRiceGenes Database

**Fig. 2:**
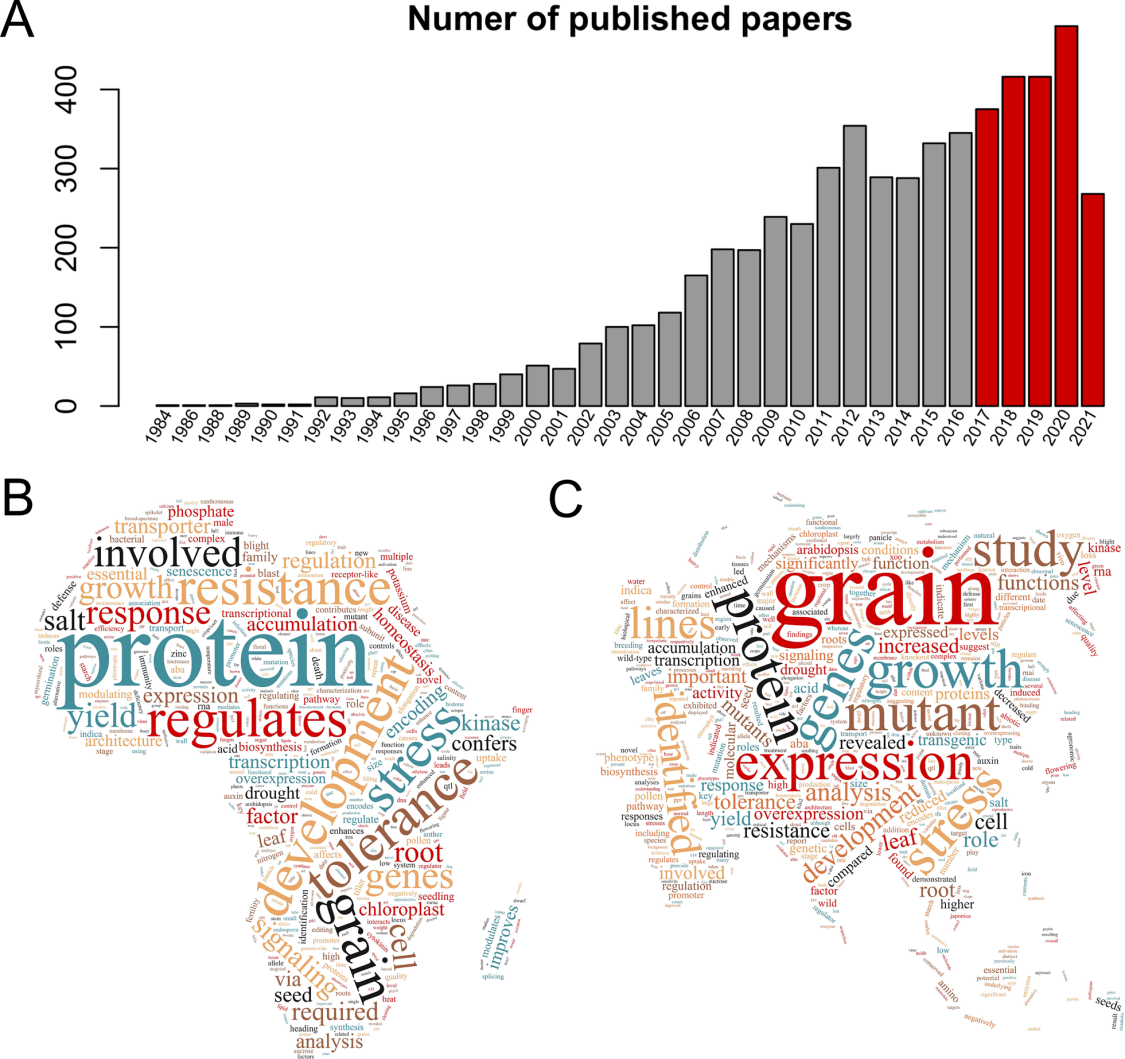
Overview of the literature on functional rice genes published in the last 5 years. **A** The number of published papers on rice functional genomics in each year. **B** A word cloud of all the abstracts of published papers on rice functional genomics since 2017. **C** A word cloud of all the titles of published papers on rice functional genomics since 2017

**Fig. 3:**
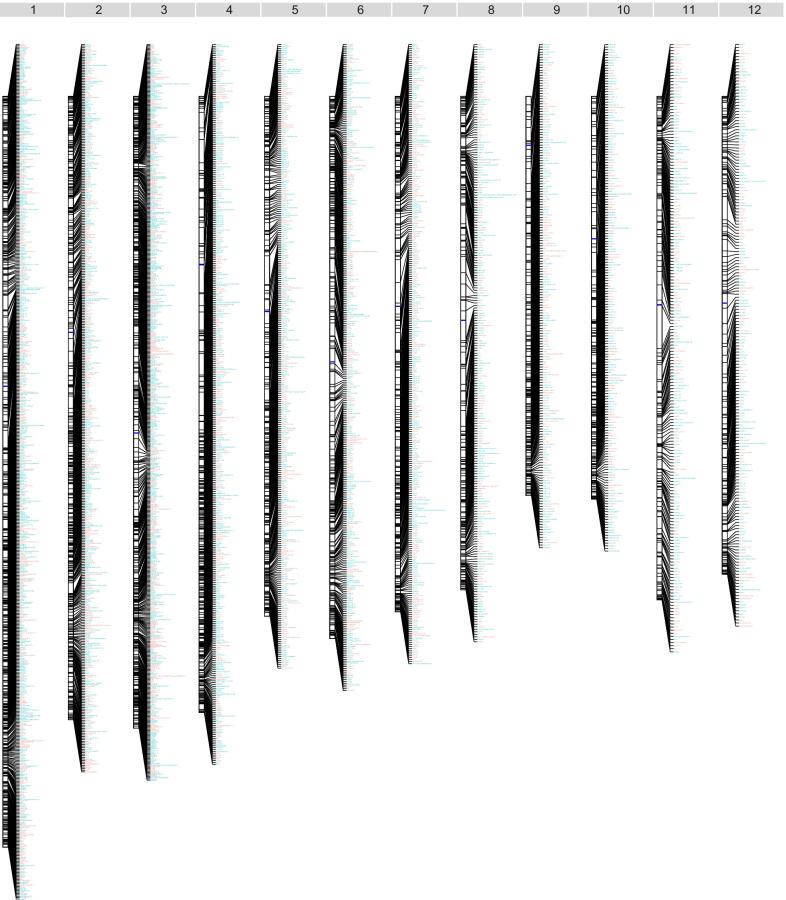
The genomic distribution of 4021 functionally characterized rice genes collected in the funRiceGenes database. Genes collected in the last 5 years are highlighted in salmon

Since being published in December 2017, the funRiceGenes database has grown by the addition of over 1300 newly cloned rice genes. More than 1950 published literature on these genes was deposited in funRiceGenes (Fig. 2A). It was found that the keywords including protein, grain, development, stress, genes, and tolerance were enriched in the titles of these literature (Fig. 2B). Similarly, the keywords including grain, protein, mutant, expression, genes, growth, and tolerance were most frequently used in the abstracts of these literature (Fig. 2C). These enriched keywords represent the research focuses on rice functional genomics studies in recent years. Among the ~ 4100 genes collected in the funRiceGenes database, 4021 genes can be anchored on the 12 chromosomes (Fig. 3) (Yu et al. 2019).

Understanding the genetic mechanisms underlying the size and weight of grains is critical to the improvement of rice yield. Up to November 2021, 162 genes regulating grain size and weight in rice were collected in funRiceGenes, including *Grain Size and Abiotic stress tolerance 1* (*GSA1*), which is a positive regulator of grain size (Dong et al. 2020). Overexpression of *GSA1* led to enhanced grain size and weight, as well as improved resistance to abiotic stresses, including high salt, drought, and high temperature. Unlike abiotic stress resistance genes, disease resistance genes usually lead to reduced yield in rice. Among all the 185 disease related genes collected in funRiceGenes, 68 genes were involved in blast disease resistance, 58 genes participated in blight disease resistance, while 9 genes were related to both diseases.

Plant hormones are biochemicals playing critical roles in the regulation of all aspects of plant growth and development, including auxin, gibberellins (GA), abscisic acid (ABA), cytokinins (CK), salicylic acid (SA), ethylene (ET), jasmonates (JA), brassinosteroids (BR), and strigolactones. A total of 623 hormone related genes were collected in funRiceGenes, including 171 auxin related genes, 142 GA related genes, 126 ethylene related genes, 105 SA related genes, 101 JA related genes, 89 BR related genes, 68 CK related genes, and 21 strigolactone related genes. Among all the 623 genes, ACE1 (ACCELERATOR OF INTERNODE ELONGATION 1) and DEC1 (DECELERATOR OF INTERNODE ELONGATION 1) are GA responsive genes identified in recent years, which act antagonistically in the regulation of internode stem elongation in rice (Nagai et al. 2020).

In the last 5 years, the funRiceGenes database has also witnessed notable progress in the identification of genes related to various phenotypic traits in rice, including asexual reproduction and herbicide resistance. BABY BOOM1 (BBM1) is an AP2/ERF transcription factor expressed in sperm cells, which can induce parthenogenesis in rice (Khanday et al. 2019). An apomixis system can be established by ectopic expression of BBM1 in the egg cell to achieve asexual reproduction of rice seeds, by replacing meiosis with mitosis. Benzobicyclon (BBC) is a β-triketone herbicide extensively used in weed control. A recently cloned gene *HIS1* (*HPPD INHIBITOR SENSITIVE 1*) encodes an oxidase that detoxifies BBC herbicides by catalyzing their hydroxylation, resulting in enhanced resistance against BBC and other β-triketone herbicides in rice, which would be useful in herbicide-resistant plant breeding (Maeda et al. 2019).

### Updated Interaction Networks of Functionally Characterized Rice Genes

**Fig. 4:**
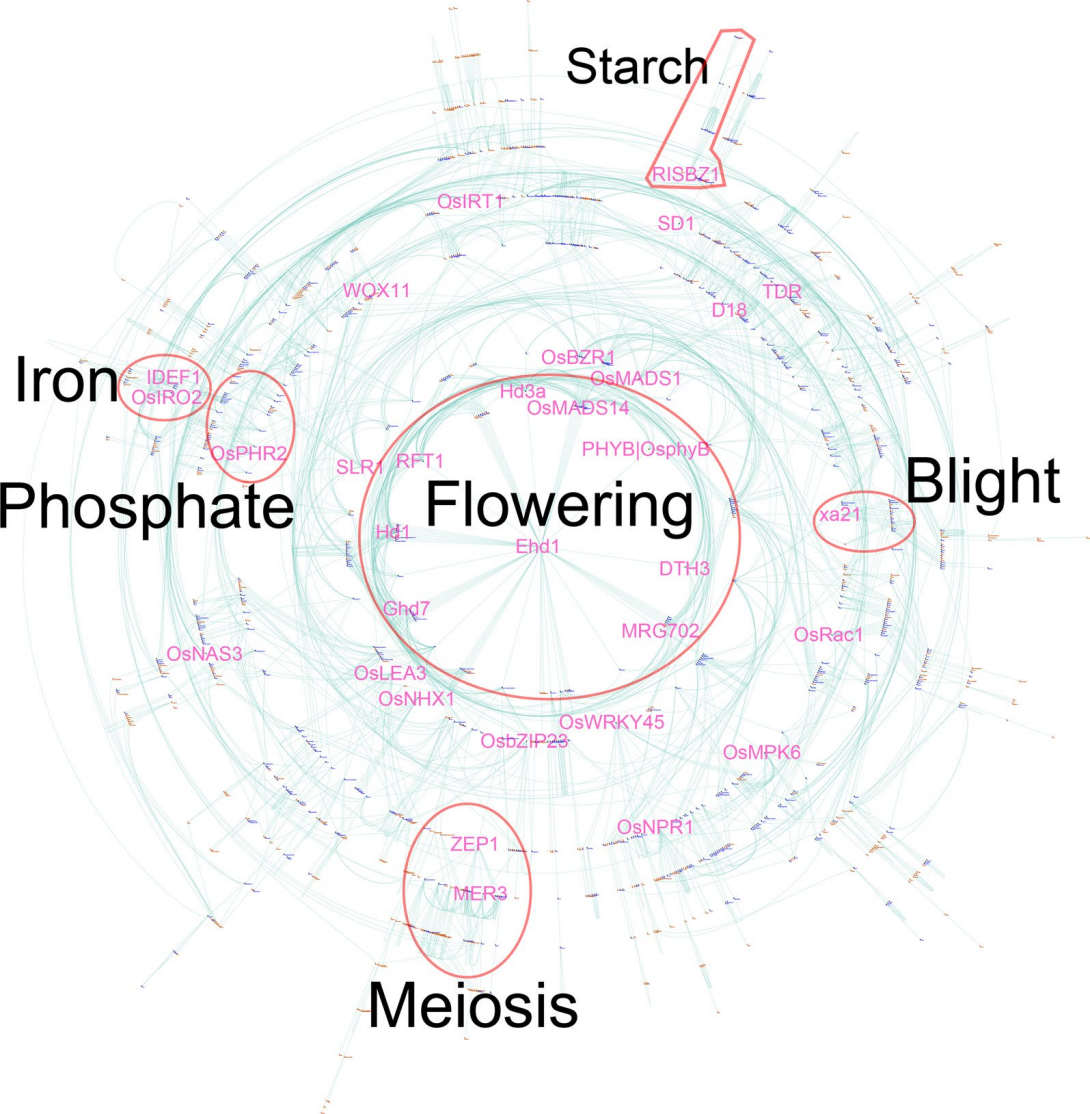
A comprehensive interaction network comprising 1281 rice genes. Each functionally characterized rice gene is represented as a node with the corresponding gene symbol marked beside. Interacting gene pairs are connected by green lines. Representative well-known genes are indicated with larger font sizes. A total of 762 genes in the largest interaction network built in the previous study are indicated in blue, while 519 new genes involved in the largest network built in the present study are in saddle brown. Genes involved in the same biological pathways are highlighted by red ellipses or polygons

Based on the ~ 4100 functionally characterized rice genes, a total of 219 interaction networks comprising 1825 genes were constructed using the approach proposed in the previous study (Yao et al. 2017). In total, 2819 connections between genes supported by 6041 pieces of evidence were extracted from the titles and abstracts of published literature. The number of genes in the largest network increased from 762 in the previous study to 1281 in the present study (Fig. 4). Accordingly, genes involved in the same biological pathway or with similar functions were found to be clustered together. We also found that genes associated with flowering are still the key component of the largest interaction network. These gene interaction networks were built by inspecting the concurrence of the symbols of two or more genes in the same sentence of published literature. The approach and the built interaction networks would shed light on future studies on gene functions in other crops.

## Conclusion

With the rapid advance of rice functional genomics studies, the funRiceGenes database was updated with more than 1300 newly cloned genes and more than 1000 members of gene families in the last 5 years. Up to November 2021, a total of ~ 4100 functionally characterized rice genes and more than 6100 gene family members were deposited in funRiceGenes, providing a valuable resource for functional dissection of genes in rice and other plants. To facilitate the utilization of funRiceGenes, we also implemented new features, including in-site searching in the static website and a BLAST interface in the interactive web application. In this study, we summarized the newly added genes and new features of funRiceGenes. The funRiceGenes database will be continuously updated with new genes and new data, severing the functional genomics researches in rice and other plants.

## Data Availability

The funRiceGenes database is freely available at https://venyao.xyz/funRiceGenes/ and https://funricegenes.github.io/.

## Abbreviations

ABA: Abscisic acid
ACE1: ACCELERATOR OF INTERNODE ELONGATION 1
BBC: Benzobicyclon
BBM1: BABY BOOM1
BR: Brassinosteroids
CK: Cytokinins
DEC1: DECELERATOR OF INTERNODE ELONGATION 1
*DEP1*: *DENSE AND ERECT PANICLE1*
ET: Ethylene
GA: Gibberellins
*GSA1*: *Grain size and abiotic stress tolerance 1*
*IPA1*: *Ideal Plant Architecture 1*
JA: Jasmonates
SA: Salicylic acid
